# Cannabis use prevalence among Baby Boomers before and after implementation of recreational retail sales in California

**DOI:** 10.1186/s13011-022-00443-9

**Published:** 2022-03-05

**Authors:** Zachary Carlson, Steven Pham, Jackie El-Sokkary, Dorie E. Apollonio

**Affiliations:** University of California San Francisco School of Pharmacy, 530 Parnassus Avenue, Suite 366, San Francisco, CA USA

**Keywords:** Cannabis, Marijuana, Legislation, Drug, California

## Abstract

**Background:**

As of 2021, 21 US states and territories allowed recreational cannabis use. Although previous research has identified an overall increase in prevalence of cannabis use after legalization, it has been less clear how this change will affect different parts of the population, including older adults, and specifically Baby Boomers, born 1946–1964, given their historically higher rates of use and a higher prevalence of comorbid conditions that could be either exacerbated or addressed by cannabis use. In this study we assessed whether implementation of recreational retail sales in California was associated with increased prevalence of cannabis use among Baby Boomers.

**Methods:**

We conducted a retrospective study of cannabis use prevalence one year before and after the implementation of recreational retail sales in California using the California Health Interview Survey (CHIS), a statewide public health surveillance dataset.

**Results:**

We found that cannabis use prevalence did not change among Baby Boomers but increased among non-Baby Boomers. Most of the factors found to be predictive of cannabis use in past research did not predict cannabis use among Baby Boomers.

**Conclusions:**

Baby Boomers did not change their consumption of cannabis in the first year after opening the retail market, despite previous research suggesting that cannabis consumption increases with access, and most previously identified predictors of use did not identify people who use cannabis in this generation. Further research is needed to determine whether these effects persist over time.

**Supplementary Information:**

The online version contains supplementary material available at 10.1186/s13011-022-00443-9.

## Highlights

- Baby Boomers, born between 1946 and 64, have unique characteristics and history that may increase their likelihood of using cannabis and health risks associated with it
- Access to recreational retail cannabis increased use in California, but not for all generational cohorts
- California Baby Boomers did not report higher use after recreational legalization and factors identified as predictors of cannabis use in past research were not associated with their use

## Introduction

Between 2012 and October 2021, 19 states in the US, along with Washington DC and Guam, legalized recreational cannabis use, [1, 2] a policy change associated with increased consumption at the population level [3–5]. Policy changes such as recreational legalization are considered to be positive social cues that are likely to increase cannabis use among adults, [6, 7] however, there has been little research assessing the effects of this normalization [7]. Although the prevalence of cannabis use is highest for younger adults, cannabis use prevalence has more than tripled among adults aged 50–64, [4, 8] and has nearly doubled among adults aged 65 years and older [9]. In Canada, nearly half (48.5%) of Baby Boomers, born between 1946 and 1964, reported using cannabis for recreational purposes only, a smaller proportion reported using both recreational and medical cannabis (19.2%), and the smallest share reported medical cannabis use only (7.1%) [10].

In the US there is widespread acceptance of cannabis use, which is generally perceived to be harmless [11]. In California, legalization of medical cannabis in 1996 was associated with greater prevalence of cannabis use by adults, including among those in the Baby Boomer generation [12–15]. Increased use in this group may be related to historical trends; people in this generational cohort were young adults and adolescents during a time when the predominant counterculture (e.g., Woodstock, “hippie culture”) accepted and arguably encouraged cannabis use [16]. People may consume cannabis in an effort to treat certain medical conditions (e.g., pain, nausea), and longitudinal research suggests that people who did not use cannabis prior to recreational legalization and who initiated cannabis use after the establishment of recreational retail sales may be seeking to treat medical conditions, potentially in response to increased ease of purchasing, the widespread availability of comparable products, and to avoid regulatory restrictions imposed on holders of medical cannabis cards (e.g., the ability to hold a commercial driver’s license, work for the federal government, and purchase firearms) [17–19]. Although medical cannabis can be less expensive than recreational cannabis, older adults in particular have reported difficulty obtaining it due to provider unwillingness to prescribe and the cost of obtaining medical cannabis cards [6, 7]. As a result, older adults have reported using recreational dispensaries to obtain medical cannabis [6].

Although cannabis may be used for medical purposes, there are also associated health risks. A 2018 systematic review found that older adults (aged 50+) that used cannabis only were significantly more likely to report major depression and serious suicidal thoughts, more likely to report other substance use and subsequent health risks attributable to substance use, and more likely to report engaging in risky behaviors, including driving under the influence (DUI) [12]. Cannabis use is associated with and may interact with physical and cognitive effects associated with aging, including fall risk, respiratory disease, cardiovascular disease, stroke, and mental health disorders such as dementia [11, 15, 20]. In addition, some research suggests people aged 65 years and older favor edibles, [21] which can contain variable and sometimes extremely high levels of THC (tetrahydrocannabinol) [22] that may lead to psychosis and could exacerbate or negatively affect the trajectory of preexisting mental illnesses such as schizophrenia [23–25].

Public health research suggests that cannabis legalization, whether recreational or medical or applicable to personal use or retail sales, has led to increased consumption [5, 26], yet more data is needed to assess the magnitude, timing, and predictors of these effects [27]. Substance use has historically declined with aging (age effects), but substance use is also driven by generational trends (cohort effects) [7, 28]. Since 1999 there have been calls for research on the prevalence of substance use among Baby Boomers as a cohort given their historically higher rates of use, the possibility of reduction in use over time due to age effects, and potential interactions with age-related health conditions [2, 7, 28, 29]. Although existing research suggests that Baby Boomer cohort effects will result in increased prevalence of cannabis use [2, 15, 28–31], models of prevalence have not previously considered the potential effects of recreational legalization in this cohort, focusing instead on medical cannabis [28, 30, 32]. Past research has noted that identifying predictors of cannabis use, which can include policy changes, is critical to developing interventions for vulnerable populations [29].

California was the first state to legalize medical cannabis use in 1996 and the effects of medical legalization were well established when the state permitted recreational use in 2016, although there was no change to the retail market until 2018. In 2018, 164 recreational retail dispensaries began selling cannabis to adults in California, and most of these dispensaries were licensed and began selling cannabis on January 1st of that year [33, 34]. After January 2018, few additional dispensaries were licensed to sell cannabis before mid-2019, [35] providing a clear demarcation of the change in access to cannabis. In this study we assessed the prevalence of cannabis use among Baby Boomers in California before and after the implementation of recreational retail cannabis sales, a policy change we anticipated would be associated with increased use due to cohort effects [5, 26]. We also assessed factors associated with cannabis use in this cohort.

## Methods

The California Health Interview Survey (CHIS) is the nation’s largest state-level health survey and is conducted using computer-assisted telephone interviews in six languages: English, Spanish, Chinese (Mandarin and Cantonese), Vietnamese, Korean, and Tagalog. Data collection relies on a random-digit-dialing (RDD) with the aim of contacting participants by 50% landline and 50% mobile phone numbers. CHIS explicitly seeks a sample that is representative of the state’s total population, estimated to be over 39 million in 2019 [36]. The survey includes all 58 California counties, and geographic stratification accounts for population size and demographics, making it possible to obtain valid estimates for smaller ethnic and racial groups [37]. CHIS data files include population weights based on the State of California Department of Finance estimates, adjusted to remove those living in group quarters, who are excluded from data collection. Each annual wave of data collection includes approximately 20,000 Californian residents. Detailed documentation on study methodology is available from the UCLA Center for Health Policy Research [37]. The survey includes questions on a range of health topics.

### Study participants and measures

All participants studied were adults (> 18 years old); we specifically considered Baby Boomers, defined as those born between 1946 and 1964, and compared them to adults in other generations.

Our three primary outcomes of interest were cannabis use, and included whether respondents had ever used cannabis, had used cannabis in the past 30 days, or had formerly used cannabis but did not currently use it. Use variables were identified from the following questions: “The next questions are about marijuana also called cannabis or weed, hashish, and other products containing THC. There are many methods for consuming these products, such as smoking, vaporizing, dabbing, eating, or drinking. (a) Have you ever, even once, tried marijuana or hashish in any form? (b) How long has it been since you last used marijuana or hashish in any form? (c) During the past 30 days, on how many days did you use marijuana, hashish, or another THC product?” We coded these variables as binary indicating that a respondent had ever used cannabis if the answer to (a) was yes and currently used cannabis if the answer to (c) was greater than zero. We defined former cannabis use to exclude “infrequent users” identified in previous research as those who might consume cannabis less often than once per year (Solowij et al., 2019); as a result, respondents were classified as having formerly used if their reported prior use of cannabis was at least 15 years ago.

We used reported year of birth to assign participants to generations (Silent Generation: 1928–45; Baby Boomer: 1946–64; Generation X: 1965–80; Millennial: 1981–96; Generation Z: 1997–2012). To assess potential predictors of cannabis use we included variables associated with cannabis use in prior research [9, 32, 38–41]. These were self-reported sex (reference category = female), race/ethnicity (Latinx, American Indian/Alaskan Native, Asian, African American, White; reference category = other), education (reference category = high school or less, some college, or college graduate; we defined “some college” as attending some college, vocational school, or attaining a two-year Associate degree), household income (ordinal variable; < $40,000, $40,000 - $80,000, $80,000 - 120,000, and > $120,000 salary), asthma diagnosis (reference category = no asthma), retired (reference category = not retired), unemployed status (yes if unemployed and looking for work; reference category = not unemployed), disabled (yes if receiving Social Security, disability, or and workers compensation in the last 30 days; reference category = not disabled), smoking history (yes if smoked > 100 lifetime cigarettes; reference category = no history of smoking), overweight status (yes if BMI ≥ 25.0; reference category = not overweight), felt nervous most or all of the past 30 days (original response categories were not at all, a little of the time, some of the time, most of the time, all of the time; an answer of either of the last two response categories indicated this category; the first three served as the reference category), felt depressed most or all of the past 30 days (original response categories were not at all, a little of the time, some of the time, most of the time, all of the time; an answer of either of the last two response categories indicated this category; the first three served as the reference category), and experienced psychological distress in the past 30 days (yes or no; reference category = no). The exact questions and answer categories underlying these variables are provided in the Supplement.

### Analytical strategy

We used code provided by CHIS to pool multiple cycles of data and create population weights accounting for the multi-year files; the concatenation for our analysis (CHIS 2017 and 2018) only involved data of the same jackknife coefficient [37]. CHIS only included questions in the 2017 and 2018 files that were asked in identical format. Although item missing rates during data collection range from 0.5 to 5.6%, variables do not contain missing values as CHIS imputes values when respondents do not provide a valid response [42]. We used population-weighted logistic regression to test the hypothesis that the population prevalence of Baby Boomers using cannabis in California would increase after implementation of recreational retail cannabis sales in 2018, relative to non-Baby Boomers. We compared differences in the prevalence of cannabis use before and after this policy change; our primary outcomes (described above) were ever use of cannabis, use in the past 30 days, and former use. We also used population-weighted multivariate logistic regression to identify whether known factors associated with cannabis use were predictive for Baby Boomers, non-Baby Boomers, and all adults sampled in both years. For the multivariate regressions we conducted sensitivity analyses by conducting analyses for each year separately as well as both years together. All statistical analyses were completed using Stata 17.

## Results

CHIS surveyed 42,330 respondents over the course of the study period: 21,153 in 2017 and 21,177 in 2018. Baby Boomers constituted 31% of the population-sample in both years. In this population-weighted sample, the Baby Boomer cohort had a higher share of American Indian/Alaska Native respondents (0.9%) relative to other generations (0.6%), a lower share of Asian American respondents (12.7% versus 15.2%), a lower share of Latinx respondents (16.1% versus 24.1%), a higher share of White respondents (53.0% versus 39.8%), and a lower share of respondents categorized as Other (10.6% versus 14.5%). Education levels were similar. The Baby Boomer cohort had a higher share of respondents with household incomes above $120,000 relative to other generations (29.1% versus 25.6%), as well as respondents with an asthma diagnosis (10.1% versus 8.8%), retirees (44.1% versus 28.7%), disabled respondents (5.9% versus 1.7%), respondents with a history of smoking (38.9% versus 30.8%), respondents who were overweight (66.3% versus 58.6%), and lower shares of respondents reporting unemployment (1.7% versus 4.7%), feeling nervous in the past 30 days (6.1% versus 9.1%) and reporting psychological distress in the past 30 days (3.7% versus 5.0%). Results are provided in Table 1.

**Table 1:** Demographic characteristics of population-weighted CHIS adult respondents (Baby Boomers^a^, non-Baby Boomers, and all adults) in 2017, 2018, and both years combined

	Baby Boomers	non-Baby Boomers	All adults
*Self-reported sex*			
Female	52.3%	50.9%	51.2%
Male	47.7%	49.1%	48.8%
*Race/ethnicity*			
American Indian/Alaska Native	0.9%	0.6%	0.7%
Asian American	12.7%	15.2%	14.7%
Black	6.6%	5.6%	5.8%
Latinx	16.1%	24.1%	22.4%
White	53.0%	39.8%	42.7%
Other	10.6%	14.5%	13.7%
*Education*			
High school or less	37.9%	38.1%	38.1%
Some college	21.9%	22.9%	22.6%
College graduate	40.1%	39.0%	39.3%
*Household income*			
<$40,000	32.3%	33.0%	32.8%
$40,000 - $80,000	22.3%	25.2%	24.6%
$80,000 - $120,000	16.2%	16.2%	16.2%
>$120,000	29.1%	25.6%	26.4%
*Other variables*			
Asthma diagnosis	10.1%	8.8%	9.1%
Retired	44.1%	28.7%	32.0%
Unemployed	1.7%	4.7%	4.1%
Disabled	5.9%	1.7%	2.6%
Smoking history	38.9%	30.8%	32.5%
Overweight	66.3%	58.6%	60.3%
Felt nervous most or all of the time in the past 30 days	6.1%	9.1%	8.5%
Felt depressed most or all of the time in the past 30 days	2.4%	2.3%	2.3%
Had psychological distress in the past 30 days	3.7%	5.0%	4.7%

^a^Baby Boomers = individuals born 1946–1964

Data source: 2017–2018 CHIS public use data files

### Differences in prevalence of use between 2017 and 2018

We used univariate logistic regressions to assess reported differences in cannabis use measures between 2017 and 2018 for the Baby Boomer cohort, non-Baby Boomer generational cohorts, and all adults together; odds ratios reference the change in prevalence from 2017 to 2018 and 95% confidence intervals are provided to identify whether changes are statistically significant, as shown in Table 2. We hypothesized that Baby Boomers would report increased cannabis use prevalence between 2017 and 2018 but found no statistically significant difference in this population-weighted sample between 2017 and 2018 for any of the reported outcome variables: having ever used cannabis [1.10 (0.75–1.58)], use in the past 30 days [1.22 (0.96–1.55)], or having formerly used cannabis [0.81 (0.66–1.01)]. In comparison, after the implementation of recreational retail cannabis sales, non-Baby Boomers were no more likely to report ever use of cannabis [1.12 (0.96–1.29)], significantly more likely to report having used cannabis within the past 30 days [1.25 (1.07–1.46)], and significantly less likely to report only former use [0.85 (0.74–0.97)]. All adults were significantly more likely to report ever use of cannabis [1.11 (1.03–1.21)], significantly more likely to report current use of cannabis [1.24 (1.09–1.42)], and significantly less likely to report only former use of cannabis [0.86 (0.77–0.96)]. The magnitude of these differences in prevalence of use between Baby Boomers and other generations ranged from 4.9 to 36.1 percentage points; 56.1% of Baby Boomers reported having ever used cannabis versus 51.2% of non-Baby Boomers, 22.1% of Baby Boomers reported having used cannabis in the past 30 days versus 33.5% of non-Baby Boomers, and 57.9% of Baby Boomers who had ever used cannabis reported only former use versus 21.8% of non-Baby Boomers.

**Table 2:** Differences in prevalence of cannabis use between 2017 and 2018 in California for Baby Boomers^a^ relative to other generations and all California residents (odds ratios drawn from univariate logistic regression)

	Baby Boomers			Non-Baby Boomers			All adults		
	OR	CI		OR	CI		OR	CI	
Ever used cannabis	1.10	(0.76–1.59)		1.12	(0.96–1.29)		1.11	(1.03–1.21)	**
Used cannabis in the last 30 days	1.22	(0.96–1.55)		1.25	(1.07–1.46)	**	1.24	(1.09–1.42)	**
Former use of cannabis	0.81	(0.66–1.01)		0.85	(0.74–0.97)	*	0.86	(0.77–0.96)	**
	Baby Boomers			Non-Baby Boomers			All adults		
	Prevalence			Prevalence			Prevalence		
Ever used cannabis	56.1%			51.2%			52.2%		
Used cannabis in the last 30 days	22.1%			33.5%			31.0%		
Former use of cannabis	57.9%			21.8%			30.2%		

^a^Baby Boomers = individuals born 1946–1964

Data source: 2017–2018 CHIS public use data files

*p < 0.05; **p < 0.01

### Predictors of use

We used multivariate logistic regression to simultaneously assess potential associations between cannabis use measures and previously identified predictors of use. We report our findings as odds ratios with 95% confidence intervals in Table 3. We did not anticipate that predictors of use would change across different years for Baby Boomers given that there were no significant differences between prevalence of use from 2017 to 2018. Nonetheless we conducted sensitivity analyses by analyzing each year separately. Given that the results were comparable for both years, our results below report results using combined 2017 and 2018 data.

**Table 3:** Factors associated with use of cannabis in California for Baby Boomers, non-Baby Boomers^a^, and all adults (odds ratios drawn from multivariate logistic regressions for combined 2017 and 2018 data)

	Ever used cannabis								
	Baby Boomers			Non-Baby Boomers			All adults		
	OR	CI		OR	CI		OR	CI	
*Self-reported sex (reference = female)*									
Male	2.03	(1.37–3.00)	**	1.56	(1.33–1.83)	**	1.12	(0.89–1.40)	
*Race/ethnicity (reference = other)*									
American Indian/Alaska Native	3.98	(1.82–8.72)	**	2.94	(1.29–6.73)	*	1.63	(0.78–3.43)	
Asian American	0.19	(0.08–0.44)	**	0.36	(0.26–0.51)	**	0.67	(0.43–1.04)	
Black	1.79	(1.00–3.22)	*	1.54	(0.72–3.31)		1.37	(0.63–2.97)	
Latinx	0.72	(0.36–1.45)		0.88	(0.64–1.21)		0.76	(0.56–1.01)	
White	2.95	(1.50–5.79)	**	1.43	(1.12–1.82)	**	1.07	(0.78–1.47)	
*Education (reference = high school or less)*									
Some college	2.06	(1.31–3.24)	**	1.91	(1.61–2.27)	**	0.98	(0.77–1.26)	
College graduate	1.70	(1.07–2.70)	*	1.68	(1.16–2.42)	**	0.79	(0.61–1.02)	
*Household income (reference = less than $40,000)*									
$40,000 - $80,000	1.12	(0.78–1.59)		1.22	(1.01–1.46)	*	0.92	(0.73–1.16)	
$80,001 - $120,000	1.15	(0.59–2.27)		1.50	(1.03–2.17)	*	0.98	(0.56–1.70)	
>$120,000	1.49	(0.77–2.87)		1.91	(1.29–2.83)	**	0.88	(0.60–1.31)	
*Other variables*									
Asthma diagnosis	1.16	(0.76–1.76)		1.49	(1.14–1.95)	**	1.17	(0.85–1.59)	
Retired	1.02	(0.59–1.76)		0.44	(0.36–0.54)	**	0.74	(0.58–0.94)	*
Unemployed	1.00	(0.46–2.18)		1.15	(0.79–1.69)		1.02	(0.62–1.67)	
Disabled	1.77	(0.84–3.72)		1.62	(0.81–3.23)		1.02	(0.56–1.88)	
Smoking history	4.41	(2.59–7.52)	**	4.02	(3.19–5.06)	**	1.34	(1.08–1.66)	**
Overweight	0.91	(0.63–1.32)		0.84	(0.69–1.03)		0.64	(0.50–0.82)	**
Felt nervous most or all of the time in the past 30 days	1.72	(0.81–3.63)		1.57	(1.18–2.09)	**	1.31	(0.93–1.85)	
Felt depressed most or all of the time in the past 30 days	1.02	(0.35–2.98)		1.21	(0.62–2.36)		0.93	(0.50–1.72)	
Had psychological distress in the past 30 days	1.00	(0.38–2.65)		1.87	(1.01–3.43)	*	2.03	(1.28–3.23)	**
	**Current cannabis use (past 30 days)**								
	Baby Boomers			Non-Baby Boomers			All adults		
	OR	CI		OR	CI		OR	CI	
*Self-reported sex (reference = female)*									
Male	1.425	(0.97–2.09)		1.069	(0.84–1.36)		1.12	(0.89–1.40)	
*Race/ethnicity (reference = other)*									
American Indian/Alaska Native	3.822	(0.95–15.32)		1.324	(0.51–3.43)		1.63	(0.78–3.43)	
Asian American	0.471	(0.12–1.78)		0.672	(0.41–1.09)		0.67	(0.43–1.04)	
Black	1.407	(0.55–3.61)		1.437	(0.63–3.27)		1.37	(0.63–2.97)	
Latinx	0.849	(0.39–1.86)		0.724	(0.53–0.99)	*	0.76	(0.56–1.01)	
White	1.509	(0.75–3.03)		1.068	(0.74–1.53)		1.07	(0.78–1.47)	
*Education (reference = high school or less)*									
Some college	0.97	(0.61–1.54)		0.983	(0.74–1.31)		0.98	(0.77–1.26)	
College graduate	0.941	(0.52–1.71)		0.764	(0.52–1.13)		0.79	(0.61–1.02)	
*Household income (reference = less than $40,000)*									
$40,000 - $80,000	1.115	(0.70–1.76)		0.857	(0.65–1.13)		0.92	(0.73–1.16)	
$80,001 - $120,000	1.275	(0.56–2.88)		0.882	(0.46–1.71)		0.98	(0.56–1.70)	
>$120,000	0.912	(0.45–1.84)		0.897	(0.52–1.54)		0.88	(0.60–1.31)	
*Other variables*									
Asthma diagnosis	1.257	(0.76–2.08)		1.137	(0.79–1.64)		1.17	(0.85–1.59)	
Retired	1.303	(0.79–2.14)		0.704	(0.50–0.98)	*	0.74	(0.58–0.94)	*
Unemployed	1.824	(0.68–4.89)		0.921	(0.53–1.60)		1.02	(0.62–1.67)	
Disabled	0.956	(0.44–2.09)		1.092	(0.41–2.89)		1.02	(0.56–1.88)	
Smoking history	1.845	(1.30–2.61)	**	1.302	(1.01–1.68)	*	1.34	(1.08–1.66)	**
Overweight	0.811	(0.57–1.15)		0.62	(0.46–0.84)	**	0.64	(0.50–0.82)	**
Felt nervous most or all of the time in the past 30 days	1.487	(0.64–3.43)		1.249	(0.87–1.79)		1.31	(0.93–1.85)	
Felt depressed most or all of the time in the past 30 days	1.192	(0.43–3.29)		0.914	(0.45–1.85)		0.93	(0.50–1.72)	
Had psychological distress in the past 30 days	1.385	(0.50–3.81)		2.106	(1.32–3.35)	**	2.03	(1.28–3.23)	**
	**Former cannabis use**								
	Baby Boomers			Non-Baby Boomers			All adults		
	OR	CI		OR	CI		OR	CI	
*Self-reported sex (reference = female)*									
Male	0.78	(0.57–1.09)		1.11	(0.77–1.59)		1.04	(0.82–1.31)	
*Race/ethnicity (reference = other)*									
American Indian/Alaska Native	0.30	(0.07–1.38)		1.00	(0.26–3.84)		0.75	(0.28–1.98)	
Asian American	1.85	(0.53–6.42)		0.54	(0.25–1.19)		0.69	(0.40–1.19)	
Black	0.80	(0.30–2.13)		0.69	(0.30–1.56)		0.87	(0.37–2.03)	
Latinx	0.94	(0.46–1.89)		1.17	(0.72–1.89)		1.01	(0.71–1.45)	
White	0.60	(0.30–1.20)		1.38	(0.86–2.23)		1.27	(0.84–1.91)	
*Education (reference = high school or less)*									
Some college	1.00	(0.63–1.57)		0.82	(0.55–1.20)		0.87	(0.66–1.15)	
College graduate	1.10	(0.59–2.05)		1.29	(0.93–1.80)		1.26	(0.90–1.78)	
*Household income (reference = less than $40,000)*									
$40,000 - $80,000	1.18	(0.79–1.76)		1.12	(0.83–1.52)		1.05	(0.83–1.32)	
$80,001 - $120,000	1.16	(0.60–2.26)		0.82	(0.45–1.48)		0.90	(0.61–1.33)	
>$120,000	0.98	(0.63–1.52)		0.98	(0.44–2.15)		1.03	(0.66–1.60)	
*Other variables*									
Asthma diagnosis	0.96	(0.59–1.56)		0.68	(0.43–1.06)		0.76	(0.58–1.00)	
Retired	0.91	(0.63–1.31)		1.91	(1.17–3.14)	*	2.06	(1.50–2.81)	**
Unemployed	0.52	(0.24–1.13)		0.81	(0.40–1.63)		0.64	(0.37–1.11)	
Disabled	1.02	(0.52–1.99)		1.93	(0.63–5.94)		1.64	(0.93–2.89)	
Smoking history	0.58	(0.42–0.80)	**	1.38	(0.87–2.19)		1.16	(0.83–1.62)	
Overweight	1.00	(0.70–1.41)		1.77	(1.12–2.77)	*	1.56	(1.23–1.99)	**
Felt nervous most or all of the time in the past 30 days	0.76	(0.33–1.78)		0.59	(0.34–1.03)		0.62	(0.44–0.88)	**
Felt depressed most or all of the time in the past 30 days	0.74	(0.26–2.10)		1.11	(0.51–2.42)		1.05	(0.60–1.86)	
Had psychological distress in the past 30 days	0.82	(0.34–2.02)		0.40	(0.18–0.91)	*	0.47	(0.25–0.91)	*

^a^Baby Boomers = individuals born 1946–1964

Data source: 2017–2018 CHIS public use data files

*p < 0.05; **p < 0.01

### Predictors of ever use of cannabis

Among those identified as Baby Boomers who had ever used cannabis, statistically significant predictors included being male [2.03 (1.37–3.00)], American Indian/Alaskan Native [3.98 (1.82–8.72)], Asian American [0.19 (0.08–0.44)], Black [1.79 (1.00–3.22)], and White [2.95 (1.50–5.79)], having some college education [2.04 (1.31–3.24)], being a college graduate [1.70 (1.07–2.70)], and smoking history [4.46 (2.59–7.52)]. There were no statistically significant associations with being Latinx [0.72(0.36–1.45)], income of $40,000–$80,000 [1.12 (0.78–1.59)], income of $80,001–$120,000 [1.15 (0.59–2.27)], income above $120,000 [1.49 (0.77–2.87)], asthma diagnosis [1.16 (0.76–1.76)], being retired [1.02 (0.59–1.76)], being unemployed [1.00 (0.46–2.18)], being disabled [1.77 (0.84–3.72)], being overweight [0.91 (0.63–1.32)], feeling nervous in the past 30 days [1.72 (0.81–3.63)], feeling depressed in the past 30 days [1.02 (0.35–2.98)], or having psychological distress in the past 30 days [1.00 (0.38–2.65)]. Results are provided in Table 3.

Among non-Baby Boomers, there were statistically significant associations with being male [1.56 (1.33–1.83)], being American Indian/Alaska Native [2.94 (1.29–6.73)], being Asian American [0.36 (0.26–0.51)], being White [1.43 (1.12–1.82)], having some college education [1.91 (1.61–2.27)], being a college graduate [1.91 (1.61–2.27)], having household income of $40,000–$80,000 [1.22 (1.01–1.46)], having household income of $80,001–$120,000 [1.50 (1.03–2.17)], having household income of more than $120,000 [1.91 (1.29–2.83)], having an asthma diagnosis [1.49 (1.14–1.95)], being retired [0.44 (0.36–0.54)], having a history of smoking [4.02 (3.19–5.06)], having felt nervous in the past 30 days [1.57 (1.18–2.09)], and having psychological distress in the past 30 days [1.87 (1.01–3.43)]. There were no statistically significant associations with being Black [1.54 (0.72–3.31)], being Latinx [0.88 (0.64–1.21)], being unemployed [1.15 (0.79–1.69)], being disabled [1.62 (0.81–3.23)], being overweight [0.84 (0.69–1.03)], or having felt depressed in the past 30 days [1.21 (0.62–2.36)].

For all adults, there were statistically significant associations with being retired [0.74 (0.58–0.94)], having a history of smoking [1.34 (1.08–1.66)], and being overweight [0.64 (0.50–0.82)]. There were no statistically significant associations with being male [1.12 (0.89–1.40)], being American Indian/Alaska Native [1.63 (0.78–3.43)], being Asian American [0.67 (0.43–1.04)], being Black [1.37 (0.63–2.97)], being Latinx [0.76 (0.56–1.01)], being White [1.07 (0.78–1.47)], having some college education [0.98 (0.77–1.26)], being a college graduate [0.79 (0.61–1.02)], having household income of $40,000–$80,000 [0.92 (0.73–1.16)], having household income of $80,001–$120,000 [0.98 (0.56–1.70)], having household income of more than $120,000 [0.88 (0.60–1.31)], having an asthma diagnosis [1.17 (0.85–1.59)], being unemployed [1.02 (0.62–1.67)], being disabled [1.02 (0.56–1.88)], having felt nervous in the past 30 days [1.31 (0.93–1.85)], having felt depressed in the past 30 days [0.93 (0.50–1.72)], or having psychological distress in the past 30 days [2.03 (1.28–3.23)].

### Predictors of cannabis use in the past 30 days

Among those identified as Baby Boomers who reported having used cannabis in the past 30 days the only statistically significant predictor of use was reported smoking [1.85 (1.30–2.61)]. There were no statistically significant associations with being male [1.43 (0.97–2.09)], being American Indian/Alaska Native [3.82 (0.95–15.32)], being Asian American [0.47 (0.12–1.78)], being Black [1.41 (0.55–3.61)], being Latinx [0.85 (0.39–1.86)], being White [1.51 (0.75–3.03)], having some college education [0.97 (0.61–1.54)], being a college graduate [0.94 (0.52–1.71)], having household income of $40,000–$80,000 [1.11 (0.70–1.76)], having household income of $80,001–$120,000 [1.27 (0.56–2.88)], having household income of more than $120,000 [0.91 (0.45–1.84)], having an asthma diagnosis [1.26 (0.76–2.08)], being retired [1.30 (0.79–2.14)], being unemployed [1.82 (0.68–4.89)], being disabled [0.96 (0.44–2.09)], being overweight [0.81 (0.57–1.15)], having felt nervous in the past 30 days [1.49 (0.64–3.43)], having felt depressed in the past 30 days [1.19 (0.43–3.29)], or having psychological distress in the past 30 days [1.39 (0.50–3.81)].

Among non-Baby Boomers, there were statistically significant associations with being Latinx [0.72 (0.53–0.99)], being retired [0.70 (0.50–0.98)], having a history of smoking [1.30 (1.01–1.68)], being overweight [0.62 (0.46–0.84)], and having psychological distress in the past 30 days [2.11 (1.32–3.35)]. There were no statistically significant associations with being male [1.07 (0.84–1.36)], being American Indian/Alaska Native [1.32 (0.51–3.43)], being Asian American [0.67 (0.41–1.09)], being Black [1.44 (0.63–3.27)], being White [1.07 (0.74–1.53)], having some college education [0.98 (0.74–1.31)], being a college graduate [0.76 (0.52–1.13)], having household income of $40,000–$80,000 [0.86 (0.65–1.13)], having household income of $80,001–$120,000 [0.88 (0.46–1.71)], having household income of more than $120,000 [0.90 (0.52–1.54)], having an asthma diagnosis [1.14 (0.79–1.64)], being unemployed [0.92 (0.53–1.60)], being disabled [1.09 (0.41–2.89)], having felt nervous in the past 30 days [1.25 (0.87–1.79)], or having felt depressed in the past 30 days [0.91 (0.45–1.85)].

Among all adults, there were statistically significant associations with being retired [0.74 (0.58–0.94)], having a history of smoking [1.34 (1.08–1.66)], being overweight [0.64 (0.50–0.82)], and having psychological distress in the past 30 days [2.03 (1.28–3.23)]. There were no statistically significant associations with being male [1.12 (0.89–1.40)], being American Indian/Alaska Native [1.63 (0.78–3.43)], being Asian American [0.67 (0.43–1.04)], being Black [1.37 (0.63–2.97)], being Latinx [0.76 (0.56–1.01)], being White [1.07 (0.78–1.47)], having some college education [0.98 (0.77–1.26)], being a college graduate [0.79 (0.61–1.02)], having household income of $40,000–$80,000 [0.92 (0.73–1.16)], having household income of $80,001–$120,000 [0.98 (0.56–1.70)], having household income of more than $120,000 [0.88 (0.60–1.31)], having an asthma diagnosis [1.17 (0.85–1.59)], being unemployed [1.02 (0.62–1.67)], being disabled [1.02 (0.56–1.88)], having felt nervous in the past 30 days [1.31 (0.93–1.85)], or having felt depressed in the past 30 days [0.93 (0.50–1.72)],

### Predictors of former use of cannabis

Among Baby Boomers who had only formerly used cannabis, the only statistically significant association was negative, with reported smoking [0.58 (0.42–0.80)]. There were no statistically significant associations with being male [0.78 (0.57–1.09)], being American Indian/Alaska Native [0.30 (0.07–1.38)], being Asian American [1.85 (0.53–6.42)], being Black [0.80 (0.30–2.13)], being Latinx [0.94 (0.46–1.89)], being White [0.60 (0.30–1.20)], having some college education [1.00 (0.63–1.57)], being a college graduate [1.10 (0.59–2.05)], having household income of $40,000–$80,000 [1.18 (0.79–1.76)], having household income of $80,001–$120,000 [1.16 (0.60–2.26)], having household income of more than $120,000 [0.98 (0.63–1.52)], having an asthma diagnosis [0.96 (0.59–1.56)], being retired [0.91 (0.63–1.31)], being unemployed [0.52 (0.24–1.13)], being disabled [1.02 (0.52–1.99)], being overweight [1.00 (0.70–1.41)], having felt nervous in the past 30 days [0.76 (0.33–1.78)], having felt depressed in the past 30 days [0.74 (0.26–2.10)], or having psychological distress in the past 30 days [0.82 (0.34–2.02)].

Among non-Baby Boomers, there were statistically significant associations with being retired [1.91 (1.17–3.14)] and being overweight [1.77 (1.12–2.77)]. There were no statistically significant associations with being male [1.11 (0.77–1.59)], being American Indian/Alaska Native [1.00 (0.26–3.84)], being Asian American [0.54 (0.25–1.19)], being Black [0.69 (0.30–1.56)], being Latinx [1.17 (0.72–1.89)], being White [1.38 (0.86–2.23)], having some college education [0.82 (0.55–1.20)], being a college graduate [1.29 (0.93–1.80)], having household income of $40,000–$80,000 [1.12 (0.83–1.52)], having household income of $80,001–$120,000 [0.82 (0.45–1.48)], having household income of more than $120,000 [0.98 (0.44–2.15)], having an asthma diagnosis [0.68 (0.43–1.06)], being unemployed [0.81 (0.40–1.63)], being disabled [1.93 (0.63–5.94)], having a history of smoking [1.38 (0.87–2.19)], having felt nervous in the past 30 days [0.59 (0.34–1.03)], having felt depressed in the past 30 days [1.11 (0.51–2.42)], or having psychological distress in the past 30 days [0.40 (0.18–0.91)].

Among all adults, there were statistically significant associations with being retired [2.06 (1.50–2.81)], being overweight [1.56 (1.23–1.99)], having felt nervous in the past 30 days [0.62 (0.44–0.88)], and having psychological distress in the past 30 days [0.47 (0.25–0.91)]. There were no statistically significant associations with being male [1.04 (0.82–1.31)], being American Indian/Alaska Native [0.75 (0.28–1.98)], being Asian American [0.69 (0.40–1.19)], being Black [0.87 (0.37–2.03)], being Latinx [1.01 (0.71–1.45)], being White [1.27 (0.84–1.91)], having some college education [0.87 (0.66–1.15)], being a college graduate [1.26 (0.90–1.78)], having household income of $40,000–$80,000 [1.05 (0.83–1.32)], having household income of $80,001–$120,000 [0.90 (0.61–1.33)], having household income of more than $120,000 [1.03 (0.66–1.60)], having an asthma diagnosis [0.76 (0.58–1.00)], being unemployed [0.64 (0.37–1.11)], being disabled [1.64 (0.93–2.89)], having a history of smoking [1.16 (0.83–1.62)], or having felt depressed in the past 30 days [1.05 (0.60–1.86)].

## Discussion

### Summary of the evidence

Although previous research has noted the overall increase in prevalence of cannabis use after legalization, it has been less clear how this change will affect different parts of the population, including older adults who face different health risks relative to younger adults due to a higher prevalence of comorbid conditions that could be either exacerbated or addressed by cannabis use. Our findings compared prevalence of cannabis use and risk factors associated with use among Baby Boomers before and after legalization of recreational commercial cannabis sales in California. We found that cannabis use prevalence did not change among Baby Boomers but increased among non-Baby Boomers. Although individuals may use cannabis for medical purposes, cannabis use in older adults is also associated with health risks [11, 12] and it is possible that increased awareness of these risks reduced the likelihood that Baby Boomers would transition to recreational cannabis. However, previous research conducted in Colorado and the San Francisco Bay Area found that Baby Boomers may preferentially purchase cannabis in recreational dispensaries for medical use, a result that is inconsistent with this interpretation [6, 7].

We also found that although many of the predictors identified in past research as associated with cannabis use were significant when considering adults overall, few predicted reported cannabis use among Baby Boomers. Despite past research identifying potential associations between cannabis use and gender, race and ethnicity, education, employment status, and existing health conditions, [9, 32, 38–41] among Baby Boomers, for the measures we considered, only a history of smoking was associated with cannabis use in the past 30 days or with former use of cannabis. It is unclear what drives these differences. Individuals categorized as Asian American in previous studies, for example, reported lower rates of cannabis use than other groups in the population, [43] which we did not observe in our sample. This finding might reflect differences among populations aggregated into the category “Asian American” that could be more apparent in California, where the share of the population represented by people typically categorized this way is relatively large.

### Strengths and limitations

Although this research relied on a large, representative sample, the survey relied on self-report by those choosing voluntarily to participate and who are accessible by telephone, and the results were not externally validated, raising the possibility that responses were inaccurate due to sampling, recall, or social desirability bias. CHIS surveys were conducted continuously throughout 2017 and 2018; as a result, some respondents had only experienced legal recreational retail cannabis sales for a brief period. The fact that almost all recreational dispensaries active in 2018 opened on January 1st mitigates this concern to some extent, nonetheless, these findings may change over time as the market becomes more established. In addition, the prevalence of cannabis use increased for non-Baby Boomers, indicating that our failure to identify an association between increased prevalence of cannabis use and recreational retail sales was specific to Baby Boomers. CHIS data consists of repeated cross-sectional surveys, meaning that we could only observe changes at the population level, rather than for specific individuals. Data limitations also meant that we could not account for every known potential predictor; this includes measures of alcohol use, which were not asked in these survey years. In addition, measures of cannabis use did not indicate mode of consumption (e.g., smoking versus edibles), dosages, or whether any or all cannabis use was prescribed by a health care provider. We combined data from different generations to serve as a comparison group for the Baby Boomer cohort. These different generations do not necessarily have the same prevalence of use or patterns of consumption. As a result, it would be inappropriate to use the results from this comparison group as predictive; for example, the observation of negative associations between retirement and cannabis use may reflect low rates of cannabis use among adults older than Baby Boomers rather than a characteristic of retirement. Finally, California legalization of recreational cannabis use proceeded in two stages, with use and personal grows legalized in 2016, and recreational retail sales legalized in 2018, meaning that if use of recreational cannabis among Baby Boomers increased after 2016, we would not have observed this shift. However, research on recreational retail sales suggests that because legalized sales increase accessibility, they have a greater effect on consumption than legalization of personal use and grows.

### Recommendations for research

This study of shifts in the prevalence of cannabis use was conducted in a single state, California, and additional research on prevalence of use after the establishment of recreational retail sales is warranted in other states. Further study could also consider whether cannabis use prevalence changes over longer periods of time, after recreational retail sales have become further normalized. Finally, additional research on perceived risks of cannabis use would help resolve questions about whether they affected Baby Boomer decision-making.

## Conclusions

Our results provide guidance for decisionmakers seeking to understand the short-term effects of establishing a recreational retail cannabis market. Existing research suggests that people in the Baby Boomer cohort were more likely to use cannabis than previous generations and has speculated that they may not show the decline in substance use prevalence previously associated with aging (age effects). We anticipated that the prevalence of cannabis use among Baby Boomers would increase in California after the implementation of recreational retail cannabis sales in 2018, consistent with previous research that has found increased prevalence of use in the population overall after recreational legalization. Despite these expectations, we did not observe this shift in prevalence for Baby Boomers even though we did observe an immediate increase in prevalence of use among adults from other generations. Given other research suggesting that Baby Boomers may at times have difficulty accessing medical cannabis for medical use and as a result seek to purchase at recreational dispensaries, this finding was unexpected [6, 7]. In addition, we found that few of the previously identified predictors of cannabis use were significant among Baby Boomers; only a history of smoking was significantly associated with likelihood of current use or former use. Overall, these findings suggest that Baby Boomers may potentially be demonstrating an age effect; unlike people in other generational cohorts, their self-reported cannabis use did not increase after adults in California were able to purchase recreational cannabis. Further research conducted over longer time periods and in other states could help identify whether this relationship persists.

## Supplementary Information

**Additional file 1..** Supplementary material. California Health Interview Survey questions. Source: California Health Interview Survey.

## Data Availability

Data are available for public download from the California Health Interview Survey at https://healthpolicy.ucla.edu/chis/Pages/default.aspx

## Abbreviations

CHIS: California Health Interview Survey
RDD: Random-digit dialing
UCLA: University of California Los Angeles
THC: Tetrahydrocannabinol
BMI: Body mass index
OR: Odds ratio
CI: Confidence interval
