# Supplementary material for: Cannabis use prevalence among Baby Boomers before and after implementation of recreational retail sales in California
Source: Subst Abuse Treat Prev Policy. 2022 Mar 5;17:17. doi: 10.1186/s13011-022-00443-9 (PMC8898516; doi:10.1186/s13011-022-00443-9)
Supplement: Supplementary file 1 — Additional file 1.. Supplementary material. California Health Interview Survey questions. Source: California Health Interview Survey. [file 13011_2022_443_MOESM1_ESM.pdf]

Cannabis use prevalence among Baby Boomers before and after implementation of recreational retail sales in California

Supplement

California Health Interview Survey questions

Source: California Health Interview Survey

The next questions are about marijuana also called cannabis or weed, hashish, and other products containing THC. There are many methods for consuming these products, such as smoking, vaporizing, dabbing, eating, or drinking.

Have you ever, even once, tried marijuana or hashish in any form?

[IF NEEDED: THC is the active ingredient in marijuana.]

□□□ 01 YES

□□□ 02 NO

□□□ -7 REFUSED

□□□ -8 DON'T KNOW

How long has it been since you last used marijuana or hashish in any form?

□□□ 01 DAYS [HR: 0-365]

□□□ 02 MONTHS [HR: 0-12]

□□□ 03 YEARS [0-99]

□□□ -7 REFUSED

□□□ -8 DON'T KNOW

During the past 30 days, on how many days did you use marijuana, hashish, or another THC product?

□□□ 01 0 DAYS

□□□ 02 1-2 DAYS

□□□ 03 3-5 DAYS

□□□ 04 6-9 DAYS

□□□ 05 10-19 DAYS

□□□ 06 20-29 DAYS

□□□ 07 30 DAYS OR MORE

□□□ -7 REFUSED

□□□ -8 DON'T KNOW

What is your date of birth?

[AA1MON] -

MONTH _____ [RANGE: 1-12]

□□□ 01 JANUARY

□□□ 02 FEBRUARY

□□□ 03 MARCH

□□□ 04 APRIL

□□□ 05 MAY

□□□ 06 JUNE

□□□ 07 JULY

□□□ 08 AUGUST

□□□ 09 SEPTEMBER

□□□ 10 OCTOBER

□□□ 11 NOVEMBER

□□□ 12 DECEMBER

[AA1DAY] -

DAY _____ [RANGE: 1-31]

[AA1YR] -

YEAR _____ [RANGE: 1907-2001]

□□□ -7 REFUSED

□□□ -8 DON'T KNOW

Are you male or female?

□□□ 01 MALE

□□□ 02 FEMALE

□□□ -7 REFUSED

Are you Latino or Hispanic?

□□□ 01 YES

□□□ 02 NO

□□□ -7 REFUSED

□□□ -8 DON'T KNOW

which one or more of the following you would use to describe yourself. Would you describe yourself as Native Hawaiian, Other Pacific Islander, American Indian, Alaska Native, Asian, Black, African American, or White?

[IF R SAYS “NATIVE AMERICAN” CODE AS “4”][IF R GIVES ANOTHER RESPONSE YOU MUST SPECIFY WHAT IT IS]

[CODE ALL THAT APPLY]

□□□ 01 WHITE

□□□ 02 BLACK OR AFRICAN AMERICAN

□□□ 03 ASIAN

□□□ 04 AMERICAN INDIAN OR ALASKA NATIVE

□□□ 05 OTHER PACIFIC ISLANDER

□□□ 06 NATIVE HAWAIIAN

□□□ -7 REFUSED

□□□ -8 DON'T KNOW

□□□ 91 OTHER (SPECIFY: _____________)

What is the highest grade of education you have completed and received credit for?

□□□ 30 NO FORMAL EDUCATION

□□□ 02 GRADE SCHOOL

□□□ 03 HIGH SCHOOL OR EQUIVALENT

□□□ 04 4-YEAR COLLEGE OR UNIVERSITY

□□□ 05 GRADUATE OR PROFESSIONAL SCHOOL

□□□ 06 2-YEAR JUNIOR OR COMMUNITY COLLEGE

□□□ 07 VOCATIONAL, BUSINESS, OR TRADE SCHOOL

□□□ -7 REFUSED

□□□ -8 DON'T KNOW (OUT OF RANGE)

What is your best estimate of your household’s total annual income from all sources before taxes in 2017?

[IF NEEDED, SAY: “Include money from jobs, social security, retirement income, unemployment payments, public assistance and so forth. Also include income from interest, dividends, net income from business, farm, or rent and any other money income.”]

[IF AMOUNT GREATER THAN $999,995, ENTER "999,995"]

$_______________ AMOUNT [HR: 0-999995]

□□□ -7 REFUSED

□□□ -8 DON'T KNOW

Has a doctor ever told you that you have asthma?

□□□ 01 YES

□□□ 02 NO

□□□ -7 REFUSED

□□□ -8 DON'T KNOW

Which of the following were you doing last week?

□□□ 01 Working at a job or business,

□□□ 02 With a job or business but not at work,

□□□ 03 Looking for work, or

□□□ 04 Not working at a job or business?

□□□ -7 REFUSED

□□□ -8 DON'T KNOW

What is the main reason you did not work last week?

[IF NEEDED, SAY: “Main reason is the most important reason.”]

□□□ 01 TAKING CARE OF HOUSE OR FAMILY

□□□ 02 ON PLANNED VACATION

□□□ 03 COULDN'T FIND A JOB

□□□ 04 GOING TO SCHOOL/STUDENT

□□□ 05 RETIRED

□□□ 06 DISABLED

□□□ 07 UNABLE TO WORK TEMPORARILY

□□□ 08 ON LAYOFF OR STRIKE

□□□ 09 ON FAMILY OR MATERNITY LEAVE

□□□ 10 OFF SEASON

□□□ 11 SICK

□□□ 91 OTHER

□□□ -7 REFUSED

□□□ -8 DON'T KNOW

Altogether, have you smoked at least 100 or more cigarettes in your entire lifetime?

□□□ 01 YES

□□□ 02 NO

□□□ -7 REFUSED

□□□ -8 DON'T KNOW

These next questions are about your height and weight. How tall are you without shoes?

[IF NEEDED, SAY: "About how tall?"]

_____ FEET

_____ INCHES

_____ METERS

_____ CENTIMETERS

□□□ -7 REFUSED

□□□ -8 DON'T KNOW

{When not pregnant, how/How} much do you weigh without shoes?

[IF NEEDED, SAY: "About how much?"]

_____ POUNDS

_____ KILOGRAMS

□□□ -7 REFUSED

□□□ -8 DON'T KNOW

About how often during the past 30 days did you feel nervous—Would you say all of the time, most of the time, some of the time, a little of the time, or none of the time?

□□□ 01 ALL

□□□ 02 MOST

□□□ 03 SOME

□□□ 04 A LITTLE

□□□ 05 NONE / NEVER

□□□ -7 REFUSED

□□□ -8 DON'T KNOW

How often did you feel so depressed that nothing could cheer you up?

[IF NEEDED, SAY: “All of the time, most of the time, some of the time, a little of the time, or none of the time?”]

□□□ 01 ALL

□□□ 02 MOST

□□□ 03 SOME

□□□ 04 A LITTLE

□□□ 05 NONE / NEVER

□□□ -7 REFUSED

□□□ -8 DON'T KNOW
